# The Role of Co-Signaling Molecules in Psoriasis and Their Implications for Targeted Treatment

**DOI:** 10.3389/fphar.2021.717042

**Published:** 2021-07-20

**Authors:** Suqing Liu, Jinhua Xu, Jinfeng Wu

**Affiliations:** Department of Dermatology, Huashan Hospital, Fudan University, Shanghai, China

**Keywords:** psoriasis, Co-stimulatory molecules, Co-inhibitory molecules, T lymphocytes, immunotherapy

## Abstract

Psoriasis is a chronic, systemic immune-mediated inflammatory disease manifesting in the skin, joint or both. Co-signaling molecules are essential for determining the magnitude of the T cell response to the antigen. According to the function of co-signaling molecules, they can be divided into co-stimulatory molecules and co-inhibitory molecules. The role of co-signaling molecules in psoriasis is recognized, mainly including the co-stimulatory molecules CD28, CD40, OX40, CD27, DR3, LFA-1, and LFA-3 and the co-inhibitory molecules CTLA-4, PD-1, and TIM-3. They impact the pathological process of psoriasis by modulating the immune strength of T cells, regulating the production of cytokines or the differentiation of Tregs. In recent years, immunotherapies targeting co-signaling molecules have made significant progress and shown broad application prospects in psoriasis. This review aims to outline the possible role of co-signaling molecules in the pathogenesis of psoriasis and their potential application for the treatment of psoriasis.

## Introduction

Psoriasis is an immune-mediated, erythematous, scaly, and chronic inflammatory skin disease that can be associated with multiple systemic diseases (Korman, 2020). The main pathological features are epidermal basal layer keratinocyte hyperproliferation, capillary dilatation, and inflammatory cells infiltration (Boehncke and Brembilla, 2018). The involvement of the immune system in psoriasis is now widely accepted (Griffiths et al., 2021). Previously, T helper type 1 (Th1) cells were considered to be the dominant cells of psoriasis, because IFN-γ and IFN-γ-producing Th1 cells were abundant in the psoriasis lesions and blood, and these Th1 cells were reduced after successful treatment (Lew et al., 2004). To date, tumor necrosis factor-alpha (TNF-α)-related and IL-23/Th17-related pathways are increasingly concerned (Li et al., 2020). Psoriasis is mainly a dendritic cell (DC) and T-cell-mediated disease with complex feedback loops from antigen-presenting cells (APCs), neutrophilic granulocytes, keratinocytes, vascular endothelial cells, and the cutaneous nervous system (Boehncke and Schön, 2015). Pathogenic T cells and innate immune systems, such as macrophages, mast cells, and granulocytes, produce IL-23 to activate Th17 cells and γδ T cells, and release several mediators, such as interferon (IFN)-γ, interleukin (IL)-17A, 17F, and 22, which induce keratinocyte proliferation and persistent chronic inflammation (Ogawa et al., 2018). In the skin inflammatory microenvironment, keratinocytes produce more IL-23 and other inflammatory factors and chemokines, thus forming an IL-23/Th17 positive feedback loop that amplifies and exacerbates the chronic inflammatory process of psoriasis. (Rendon and Schäkel, 2019).

The classical two-signal hypothesis posited that both antigen and secondary stimuli are required for T cell activation (Smith-Garvin et al., 2009; Fontana and Vance, 2011). Stimulation of the T cell receptor (TCR) by major histocompatibility complex (MHC)-peptide molecules provides a preliminary signal for lymphocyte activation, known as an antigenic stimulus signal. The second signal is generated by the interaction of T cells with multiple pairs of co-signaling molecules on the surface of APCs, which is essential for determining the magnitude of the T cell response to the antigen (Baxter and Hodgkin, 2002). The interactions of co-signaling molecules in immune responses are substantially more complex than two-signal hypothesis. For instance, the combination of CD40 and CD40L can make APC express more CD80 and CD86 molecules, while CD28 can up-regulate the expression of CD40L on T cell surface, which cooperatively drives the activation of immature T cells (Edner et al., 2020). Zhu et al. proposed a tidal model that defines the primary signal as the initiator of specific immune cells reacting to extracellular stimuli. Meanwhile, the co-signals, either co-stimulatory or co-inhibitory signals, are modulators that decide the direction and magnitude of the immune response (Zhu et al., 2011). In addition to regulating the strength of immunity, some co-signaling molecules can also regulate the secretion of cytokines, the function of Th cells or the differentiation of Tregs (Chambers and Allison, 1999; Bour-Jordan and Bluestone, 2009; Podojil and Miller, 2009). Co-signaling molecules are closely related to many autoimmune diseases, such as psoriasis, systemic lupus erythematosus (SLE), rheumatoid arthritis (RA), multiple sclerosis (MS), and type 1 diabetes (Zhang and Vignali, 2016).

**FIGURE 1:**
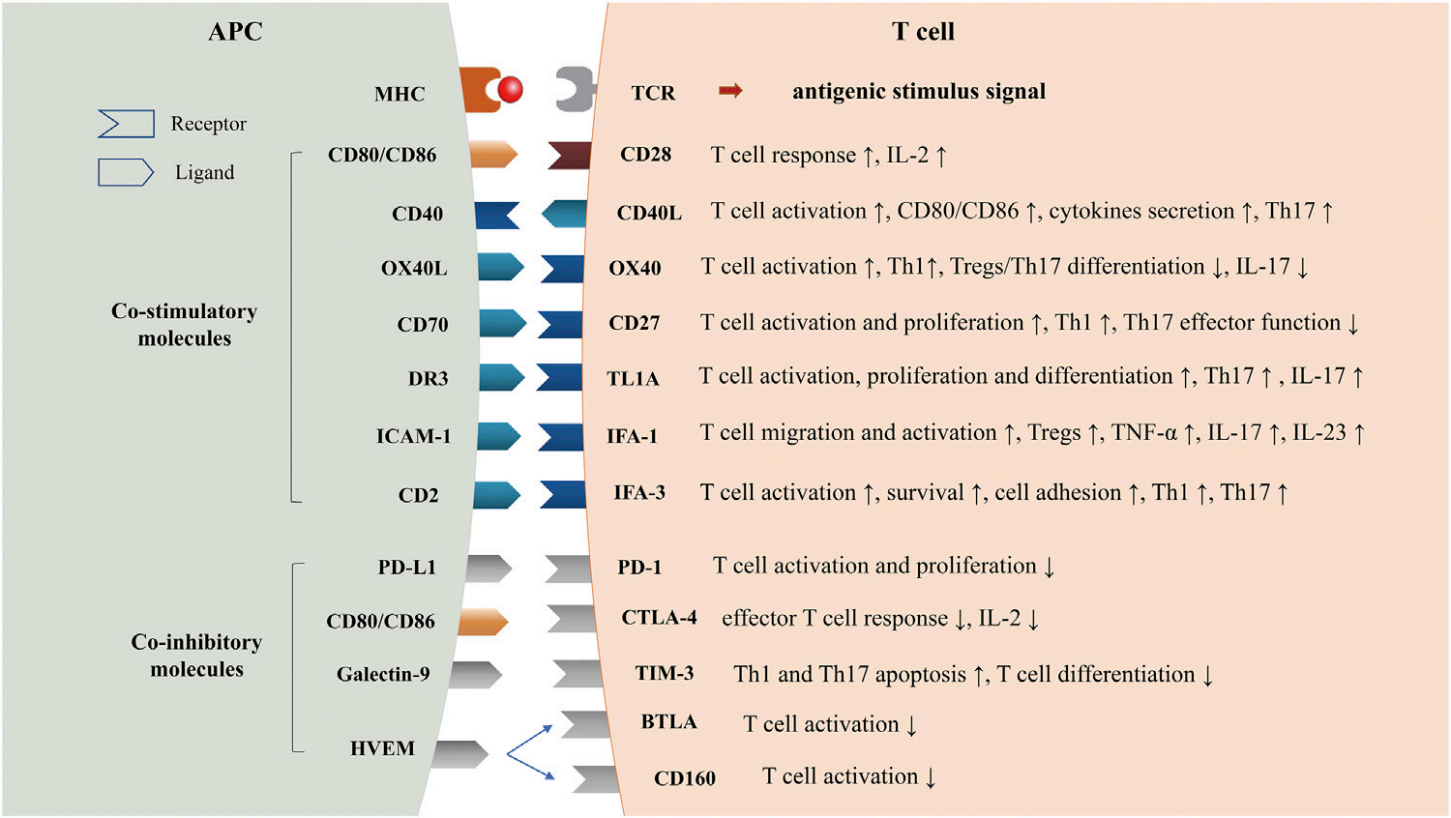
Co-stimulatory and Co-inhibitory Molecules in Psoriasis. TCR, T cell receptor; MHC, major histocompatibility complex; CD40L, CD40 ligand; TL1A, TNF-like molecule 1A; DR3, Death receptor3; LFA-1, Lymphocyte function-associated antigen-1; ICAM-1, intercellular cell adhesion molecule-1; LFA-3, Lymphocyte function-associated antigen-3; PD-1, programmed cell death one; CTLA-4, cytotoxic T lymphocyte associated protein four; TIM-3, T cell immunoglobin and mucin domain-3; HVEM, herpes virus-entry mediator; BTLA, B-lymphocyte and T-lymphocyte attenuator.

In the past decade, several biologics that are primarily aimed at inhibiting TNF-α, blocking IL-12, and IL-23, or interfering with Th17 cell development have been approved for the treatment of psoriasis (Mahil et al., 2016). Although biologics show better efficacy than conventional systemic drugs and a good safety profile with only a small increase in opportunistic infections (Di Altobrando et al., 2020; Strober et al., 2020), more clinical trials still need to be conducted to assess the long-term efficacy and side effects. In recent years, co-signaling molecules in immune cells have been reported to participate in the pathogenesis of psoriasis and provide prospects for new treatment (Edner et al., 2020; Fu et al., 2020). For instance, alefacept has been approved for treatment of moderate-to-severe psoriasis. Other targeted co-signaling molecules biologics such as KHK4083 have entered clinical trials. This review aims to outline the possible role of co-signaling molecules in the pathogenesis of psoriasis (Figure 1) and their potential application for the treatment of psoriasis (Table 1).

**TABLE 1:** Biologic drugs and therapies targeting co-signaling molecules for psoriasis treatment.

Biologics	Type	Targeting	Stage	Efficacy	Adverse event	Refs
FR255734	mAb	CD28	Preclinical	Epidermis thickness↓, inflammatory infiltration↓	/	Raychaudhuri et al. (2008)
FR104	mAb	CD28	Preclinical	Skin erythema↓,	/	Poirier et al. (2016a)
				Thickening and desquamation↓,		
				T lymphocytes and macrophages infiltration↓		
Bleselumab	mAb	CD40	Phase I	Ineffective	Transient elevations of liver transaminase enzymes	Anil Kumar et al. (2018)
KHK4083	mAb	OX40	Phase I	PASI scores↓	Mild or moderate chills, infusion site reactions	Papp et al. (2017)
Efalizumab	mAb	LFA-1	Approved 2003	/	Progressive multifocal leukoencephalopathy	(Kuschei et al., 2011; Hsu and Tsai, 2020)
			Withdrawn 2009			
Alefacept	Fusion Protein	CD2	Approved 2003	PASI scores↓↓, effector memory T cells↓, activated dendritic cells↓, inflammatory genes↓	/	(Goedkoop et al., 2004; Chamian et al., 2005, 2007; Sugiyama et al., 2008)
Siplizumab	mAb	CD2	Preclinical	Ineffective	/	(Bissonnette et al., 2009; Langley et al., 2010)
PD-L1-Fc	Fusion Protein	PD-1	Preclinical	Epidermis thickness↓, disease activity↓↓	/	Kim et al. (2016)
Abatacept (CTLA-4 Ig)	Fusion Protein	CD80, CD86	Phase III	Modest impact on psoriasis lesions, significant improvement in PsA	Increased risk for serious infection and autoimmune disorders	(Mease et al., 2011, 2017; Strand et al., 2018)
sGal-9	Stable form of galectin-9	TIM-3	Preclinical	Epidermal thickness↓↓, inflammatory infiltration↓↓, disease activity↓↓, STAT3 expression↓	/	Niwa et al. (2009)

## Co-Stimulatory Molecules

### The CD28:B7 Pathway

The CD28-B7 family might be the main co-signaling molecule in naïve T cells (Nagai and Azuma, 2019a). CD28 is an originator of co-stimulatory molecules that amplify TCR signals, induce T cell proliferation, and produce IL-2 (Aruffo and Seed, 1987). B7-1/CD80 and B7-2/CD86 are the two major ligands of CD28. CD80 is primarily expressed as a dimer, whereas CD86 is expressed as a monomer on the surface of APCs (Nagai and Azuma, 2019b).

#### The Role of the CD28:B7 Pathway in Psoriasis

Previous studies have shown that the CD28:B7 pathway plays a critically important role in the pathogenesis of many autoimmune disease including psoriasis (Daikh et al., 1997; Nagai and Azuma, 2019b). Ferenczi et al. found that epidermal T cells from skin lesions expressed high levels of the T cell co-stimulatory molecule CD28 (Ferenczi et al., 2000). Nguyen et al. evaluated the proportion of intermediate monocytes with CD86 expression in 43 patients with psoriasis and found that the upregulated expression of CD86 on the intermediate monocyte subset was positively correlated with clinical severity as measured by the psoriasis area and severity index (PASI) scores and serum beta defensin-2 (BD-2) levels (Nguyen et al., 2018). Moreover, Lima et al. observed the frequency of *ex vivo* CD4^+^ CD28^null^ cells is negatively correlated with psoriasis severity. And after clinical remission in nine patients, *ex vivo* CD4^+^ CD28^null^ lymphocytes expressing cytotoxic granules were decreased (Lima et al., 2015).

#### Targeting the CD28:B7 Pathway for the Treatment of Psoriasis

##### FR255734

Raychaudhuri et al. demonstrated that FR255734, a humanized, Fc-silent, anti-CD28 antibody, effectively inhibited T cell activation by blocking CD28/B7 co-stimulatory interactions and improved the thickness of epidermis and reduction in lymphocytic infiltration in a mouse psoriasis model (Raychaudhuri et al., 2008).

##### FR104

FR104 is a monovalent humanized Fab’ antibody fragment antagonist of CD28 that was pegylated to prolong its half-life, under development for the treatment of transplant rejection and autoimmune diseases (Poirier et al., 2012). Poirier et al. followed up to sixty-four healthy subjects for a maximum of 113 days. Overall, selective blocking of CD28 by FR104 is safe and well-tolerated (Poirier et al., 2016a). FR104 significantly reduces skin inflammation induced by aldara in non-human primates, such as skin erythema, thickening and desquamation, and prevents T lymphocytes and macrophages infiltration (Poirier et al., 2016b).

### The CD40:CD40L Pathway

CD40, a co-stimulatory receptor molecule, belongs to the TNF receptor superfamily. CD40 is mainly expressed in immune cells (B cells, APCs, and mast cells), some non-immune cells (myofibroblasts, fibroblasts, epithelial, and endothelial cells) and tumors. It binds to CD40 ligand (CD40L, CD154) expressed transiently on T cells and non-immune cells under inflammatory conditions (Chand Dakal et al., 2019). On the one hand, the interaction of CD40 and CD40L promotes APC activation and the expression of CD80/CD86 and the secretion of cytokines. On the other hand, it promotes T cell activation. The binding of CD40 and CD40L is also one of the most important second signals for B cell activation and plays an important role in B cell differentiation, maturation and antibody production (Laman et al., 2017).

#### The Role of the CD40:CD40L Pathway in Psoriasis

Lezzi et al. reported that CD40-deficient DCs exhibited reduced cytokines release and failed to drive Th17 development *in vitro*. Their data demonstrated that CD40-CD40L cross-talk integrated strong antigenic signals and microbial stimuli to induce the development of IL-17-producing CD4^+^ T cells (Iezzi et al., 2009). In psoriatic skin lesions, the number of CD40^+^ cells (keratinocytes, Langerhans cells, mature DCs) and CD40L^+^ mast cells is higher than that in healthy skin (Haimakainen et al., 2017). In patients with psoriatic arthritis (PsA), the expression of CD40 was increased in synovial fluid B cells (Armas-Gonzalez et al., 2015) and the expression of CD40L was significantly upregulated on activated T cells compared to healthy controls (Daoussis et al., 2007).

#### Targeting the CD40:CD40L Pathway for the Treatment of Psoriasis

Bleselumab (ASKP1240) is a fully human IgG4 monoclonal antibody (mAb) that targets CD40 (Vincenti et al., 2020). Anil Kumar et al. evaluated the pharmacokinetics, efficacy, safety, and tolerability of bleselumab in patients with psoriasis. As compared to CD40L-specific mAbs, no clinically malignant events have been reported with bleselumab, and only a limited number of patients have transient elevations in liver transaminase enzymes. However, bleselumab did not improve the PASI scores of psoriasis (Anil Kumar et al., 2018). Due to the small sample size and the variation in some baseline patient characteristics, further clinical trials are necessary to verify the efficacy of bleselumab.

### The OX40:OX40L Pathway

OX40 (also known as ACT35, CD134, and TNFRSF4) is a co-stimulatory receptor molecule that belongs to the TNF receptor superfamily. It is mainly expressed in activated T cells. The ligand of OX40 (OX40L, also known as gp34, CD252, and TNFSF4) is a type II glycoprotein that has a TNF homology domain. OX40 and OX40L interactions play essential co-stimulatory roles in many aspects of immunity involving direct cell-cell communication (Croft, 2010; Webb et al., 2016). Regarding CD4^+^ T cell subsets, OX40 and OX40L interactions can enhance the Th1-mediated immune response, augment follicular helper T cell (Tfh) development, and antagonize Treg generation and Treg-mediated immune suppression. For CD8^+^ T cell subsets, OX40 promotes the survival and expansion of CD8^+^ T cells and the recall response of CD8^+^ memory T cells *in vivo* (Fu et al., 2020).

#### The Role of the OX40:OX40L Pathway in Psoriasis

The existing evidence indicates that OX40 suppresses the differentiation and activity of Tregs and can attenuate Th17 differentiation (Remedios et al., 2018). Li et al. found that OX40 inhibited IL-17 expression and Th17 cell-mediated autoimmunity by inducing repressive chromatin modifications at the Il17 locus by activating histone methyltransferases (Xiao et al., 2016). Interestingly, OX40 can also downregulate CTLA-4 expression (Prell et al., 2003), promote cytokines production and play a vital role in maintaining or promoting the T cell response (Croft et al., 2009). From this point of view, it might aggravate the development of psoriasis. Therefore, the effects of OX40 signaling in psoriasis are complex and need to be further explored. Several studies have shown an obviously higher level of OX40L in serum from patients with psoriasis compared with that in healthy controls, and the number of OX40^+^ cells in psoriasis lesions is also increased (Ilves and Harvima, 2013; Guo et al., 2019). These results suggest that the OX40:OX40L pathway might have obvious influence on T cell activation in psoriasis.

#### Targeting the OX40:OX40L Pathway for the Treatment of Psoriasis

KHK4083 is a fully human monoclonal antibody against OX40. In a phase I study, KHK4083 showed good efficacy at the highest dose (10 mg/ kg) in patients with mild to moderate plaque psoriasis, and it was safe and well tolerated (Papp et al., 2017). Further clinical trials are needed to evaluate the efficacy and safety of KHK4083 in a larger patient cohort.

### The CD27:CD70 Pathway

CD27 is a TNF receptor superfamily member expressed uniformly in naive T cells and selective memory T cell subsets. Its ligand, CD70, is expressed in activated APCs and some in cases on activated lymphocyte subsets (Burchill et al., 2015). CD27-CD70 binding can induce T cell activation, promote T cell survival and proliferation, increase the number of Th1 cells and break immune tolerance. Soluble CD27 (sCD27) is capable of disrupting membrane-associated CD27 ligation and signaling and inhibits effective T cell function (Burchill et al., 2015). Coquet et al. reported that CD27 signaling represses IL-17 and the chemokine receptor CCR6 expression via the c-Jun N-terminal kinase (JNK) pathway and induces specific epigenetic and transcriptional changes in differentiating Th17 cells (Coquet et al., 2013).

#### The Role of the CD27:CD70 Pathway in Psoriasis

Previous studies have shown that the levels of sCD27 were increased in the peripheral blood of patients with psoriasis and can be used to monitor disease activity (de Rie et al., 1991). A recent study demonstrated that the percentage of CD19^+^CD27^+^CD24 (high) memory regulatory B cells was inversely correlated with the severity of psoriasis (Mavropoulos et al., 2017). Remedios et al. reported that the expression of CD27 was inversely correlated with Treg IL-17 production in lesioned skin biopsies from patients with psoriasis (Remedios et al., 2018). To date, there are no drugs on targeting the CD27:CD70 pathway for the treatment of psoriasis.

### The TNF-like Molecule 1A (TL1A):Death Receptor 3 Pathway

TL1A (also known as TNFSF15 and VEGI-251) is a cytokine of the TNF family and primarily expressed by APCs, which has a membrane-bound and a soluble form (Migone et al., 2002). DR3 (TNFRSF25, APO3, LARD, TRAMP, WSL-1) which belongs to TNF receptor superfamily is the primary activating receptor of TL1A and mainly expressed on leukocytes especially on activated lymphocytes (So and Ishii, 2019). The interaction of TL1A:DR3 mediates NF-kB, mitogen-activated protein kinase (MAPK), and caspase signaling that regulates T cell activation, proliferation, differentiation and Treg cells activation and function, but also modulates apoptosis in immune cells (Ogawa and Abe, 2019; Lubrano di Ricco et al., 2020; Rodriguez-Barbosa et al., 2020).

#### The Role of the TL1A:DR3 Pathway in Psoriasis

TL1A:DR3 pathway plays an important role in promoting Th17 cell function and Th17-mediated autoimmune disease (Pappu et al., 2008; Li et al., 2014). Pappu et al. found that DR3 is selectively elevated in Th17 cells, and TL1A can promote the proliferation of effector Th17 cells. TL1A^−/−^ DCs exhibited a reduced capacity in supporting Th17 differentiation and proliferation (Pappu et al., 2008). Besides, soluble TL1A synergized with IL-23 to stimulate peripheral blood mononuclear cells (PBMCs) from patients with psoriasis to produce IL-17 (Li et al., 2014). Previous studies have shown that both protein expressions and mRNA transcripts of TL1A and DR3 were increased in psoriatic lesions (Bamias et al., 2011). Serum TL1A levels were significantly elevated in patients with psoriasis but not in patients with atopic dermatitis and health control, and the high serum TL1A levels were decreased after treatment (Li et al., 2014; Pedersen et al., 2015). Li et al. detected the expression of DR3 in PBMCs of patients with psoriasis and found that there was a positive correlation between the percentage of DR3^+^ CD8^+^ and DR3^+^ CD14^+^ cells and the PASI scores in patients with psoriasis (Li et al., 2018). That indicates the percentage of DR3^+^ CD8^+^ and DR3^+^ CD14^+^ cells might be a novel biomarker in evaluating the severity of psoriasis. Kepiro et al. report that the rs6478109 SNP of TL1A gene might be a genetic risk factor in psoriasis, while Haplotype C might be protected against psoriasis in the Hungarian population (Képíró et al., 2014). TL1A:DR3 pathway may be a promising therapeutic target of psoriasis, but there were no reports about relevant targeted drugs applicated for the treatment of psoriasis.

### The LFA-1:ICAM-1 Pathway

Lymphocyte function-associated antigen-1 (LFA-1, CD11a/CD18), a co-stimulatory molecule that belongs to the integrin superfamily, is expressed on both T cells and DCs (Simon et al., 1991). LFA-1 has important functions in T cell immunity. It can bind to intercellular adhesion molecules (ICAMs) to make LFA-1-positive cells migrate into tissues or interact with ICAM-1-positive DCs, which promote the migration and activation of T lymphocytes (Marlin and Springer, 1987). LFA-1 is essential for the proper development and function of Tregs, and when it is absent, the propensity for autoimmunity is increased (Reina and Espel, 2017).

#### The Role of the LFA-1:ICAM-1 Pathway in Psoriasis

Adhesion molecules have been shown to play important roles in the development of psoriasis. The expression of ICAM-1 is not only intensely and locally increased in endothelial and lymphocytic cells in psoriatic lesions (Cabrijan et al., 2009) but also directly related to the severity of psoriasis (Bressan et al., 2018). Mitsui et al. assessed the role of ICAM-1 and L-selectin in the psoriasiform skin of mice and found that the disease severity was significantly reduced in ICAM-1^−/−^ or L-selectin^−/−^ mice compared with wild-type mice, while it was exacerbated in L-selectin/ICAM-1^−/−^ mice, and the levels of cutaneous IL-17A, IL-23, and TNF-α were also increased in L-selectin/ICAM-1^−/−^ mice. Although ICAM-1 and L-selectin positively regulated the psoriasiform inflammation, deleting both L-selectin and ICAM-1 simultaneously induced refractory skin inflammation (Mitsui et al., 2015). This might be due to the compensatory upregulation of other adhesion molecules.

#### Targeting the LFA-1:ICAM-1 Pathway for the Treatment of Psoriasis

Efalizumab, a humanized monoclonal antibody against LFA-1, has shown a positive response to psoriasis through potently inhibiting the proliferation and cytokine production of human T cells by downregulating the co-stimulatory molecules ICOS, OX40, CD27, and 4-1BB (Kuschei et al., 2011). However, it was voluntarily withdrawn from the US market due to the risk of progressive multifocal leukoencephalopathy in 2009 (Hsu and Tsai, 2020). The side effects of LFA-1 antibodies are probably not rare because using antibodies against LFA-1 such as efalizumab can downregulate multiple cell-surface molecules, which might be involved in T cell activation, including CD3, TCR, CD4, CD8, CD28, and the integrin VLA-4 (Guttman-Yassky et al., 2008; Grönholm et al., 2016). Further studies should be carried out to develop specific pharmaceuticals with higher specificity to LFA-1. In addition, several studies have shown that some natural products, such as glycyrrhizin and gambogic acid, could improve psoriasis by inhibiting the expression of ICAM-1. Further clinical studies are needed to assess the efficacies and safeties of these natural products (Wen et al., 2014; Xiong et al., 2015).

### The LFA-3:CD2 Pathway

Lymphocyte function-associated antigen-3 (LFA-3, CD58), a co-stimulatory molecule belongs to integrin superfamily, is expressed on the surface of T cells, B cells, thymic epithelial cells and APCs (Krensky et al., 1983). It can bind to CD2, a transmembrane glycoprotein of IgSF, which is expressed on the surface of NK cells, T cells, thymocytes and DCs (Bachmann et al., 1999). The combination of LFA-3 and CD2 plays a vital role in cell adhesion, thymocyte development, memory/effector T cell activation, T cell survival and reversal of T cell anergy (Denning et al., 1987; Bierer and Hahn, 1993; Binder et al., 2020).

#### The Role of the LFA-3:CD2 Pathway in Psoriasis

Recent study has shown that keratinocytes transmit signals through the co-stimulating receptors LFA-3:CD2 and LFA-1:ICAM-1 interactions, which initiated STAT1 signaling and IFN-γ production in T cells, generated a micromilieu that enables Th1 and Th17 polarization independent of the presence of DCs (Orlik et al., 2020). Consequently, modulating keratinocyte-mediated activation of T cells directly in the skin may represent a potential strategy for the treatment of psoriasis (Binder et al., 2020).

#### Targeting the LFA-3:CD2 Pathway for the Treatment of Psoriasis

##### Alefacept

Alefacept, a LFA-3/IgG1 fusion protein that binds to CD2, is the first biologic agent approved by the US Food and Drug Administration (FDA) for the treatment of moderate-to-severe psoriasis in 2003 (Sugiyama et al., 2008). Alefacept reduced the effector memory T cells, activated DCs, and inflammatory genes in psoriatic epidermis lesion (Goedkoop et al., 2004; Chamian et al., 2005; Chamian et al., 2007). For patients who received one course of alefacept, 28% of patients achieved a PASI score of 75. For patients who received two courses of alefacept, 40 and 71% of patients respectively achieved 75 and 50 PASI score (Krueger et al., 2002; Sugiyama et al., 2008). However, some patients show little or no benefit with alefacept (Haider et al., 2007). Overall, alefacept has good efficacy and is safe and well-tolerated for moderate-to-severe psoriasis (Scheinfeld, 2005). For patients who responded, additional courses of alefacept can help achieve long-term control of plaque psoriasis, while maintaining the safety profile (Roberts et al., 2010).

##### Siplizumab

Siplizumab, a humanized anti-CD2 monoclonal antibody, may prevent T cells activation and elicit a state of alloantigen-specific unresponsiveness (Langley et al., 2010). According to two randomized, double-blind, placebo-controlled studies, siplizumab exhibited an acceptable safety profile but not yield a therapeutic benefit (Bissonnette et al., 2009; Langley et al., 2010).

## Co-Inhibitory Molecules

### The PD-1:PD-L1/PD-L2 Pathway

PD-1 (CD279), a co-inhibitory molecule on the surface of T cells, binds to its ligands PD-L1 (B7-H1, CD274) and PD-L2 (B7-DC, CD273) and inhibits the proliferation and activation of lymphocytes, maintaining T cell homeostasis (Ishida et al., 1992). It plays a vital role in the induction and maintenance of peripheral tolerance (Chamoto et al., 2017).

#### The Role of the PD-1:PD-L1/PD-L2 Pathway in Psoriasis

Bonigen et al. described that 21 cases of patients with lung cancer developed or aggravated psoriasis after anti-PD1 immunotherapy treatment. (Bonigen et al., 2017). In addition, Niu et al. found that the content of CXCR5^+^PD-1^+^ Tfh cells in the peripheral blood of Chinese patients with psoriasis was significantly decreased and positively correlated with the disease duration (Niu et al., 2015). Shin et al. evaluated blood Tfh cells in Korean patients with psoriasis and reached the same conclusion, and they added this T cell subset was not correlated with PASI scores (Shin et al., 2016). In patients with PsA, the percentages of CD4^+^PD-1^+^ and CD8^+^PD-1^+^ T cells were also significantly lower than that in healthy controls (Bartosińska et al., 2017). The reduction in PD-1 expression on T cells seems to be reasonable, since without the negative regulatory role of PD-1, the sustained activation of T cells will lead to chronic cytokine production to promote the development of psoriasis. Meanwhile, Kim et al. reported that PD-1 was overexpressed in IL-17A^+^-producing γδ T cells in imiquimod-induced psoriasis mice and psoriasis lesions from patients (Kim et al., 2016).

#### Targeting the PD-1:PD-L1/PD-L2 Pathway for the Treatment of Psoriasis

It has been reported that PD-L1-Fc inhibits anti-CD3-induced IL-17A production in CD27^–^Vγ1^–^ γδ T cells and shows great potential for the treatment of psoriasis in animal experiments (Kim et al., 2016). Kim et al. treated psoriasis-like mice with PD-L1-Fc, anti-p40 and both. Anti-p40 is an approved drug for psoriasis treatment, which can inhibit IL-23A-induced IL-17A production through binding to the p40 subunits of IL-23 and IL-12 (Lebwohl et al., 2015). They found that the reduction in epidermal thickness and disease activity in PD-L1-Fc alone group is not as significant as that of anti-p40 alone group, while the therapeutic effects of combined anti-p40 and PD-L1-Fc can be cumulative, which may be ascribed to targeting distinct IL-17-secreting γδT cell populations (Kim et al., 2016). However, due to the different T cell subsets producing IL-17 in mice and humans, further studies are necessary to prove the effectiveness of the PD-L1 fusion protein in human psoriatic lesions.

### The CTLA-4:B7 Pathway

CTLA-4 shares homology with CD28, and these two molecules compete with each other in binding to their ligands, B7 family molecules (Linsley et al., 1991). CTLA-4 has a stronger binding affinity to these ligands than CD28, leading to the suppression of effector T cell responses (Linsley et al., 1991). After T cell activation, CTLA-4 is significantly induced, while CD28 is down-regulated by internalization. CTLA-4 binds to CD80 and CD86 on DCs to induce the expression of indoleamine 2,3-dioxygenase (IDO), which then inhibits T-cell function through tryptophan deprivation (Hosseini et al., 2020).

#### The Role of the CTLA-4:B7 Pathway in Psoriasis

Although previous studies observed elevated serum concentrations of soluble CTLA-4 in patients with psoriasis (Luszczek et al., 2006), there are no correlation between the polymorphisms in the CTLA-4 gene and psoriasis in Korean and Japanese (Kim et al., 2003; Tsunemi et al., 2003). However, a study of Polish Caucasians showed that the haplotype +49G, CT60G was significantly less frequent in the psoriasis vulgaris patient group with disease onset between the ages of 21 and 40 years than that in controls and the other patient groups (Łuszczek et al., 2008). In addition, Liu et al. reported that membrane CTLA-4 (mCTLA-4) expression in the skin lesions of patients with mild psoriasis was significantly higher than that in patients with moderate and severe psoriasis. This might indicate that the expression of mCTLA-4 in skin lesions was inversely correlated with the severity of psoriasis (Liu et al., 2018).

#### Targeting the CTLA-4:B7 Pathway for the Treatment of Psoriasis

Abatacept (BMS-188667C) is a soluble, fully human fusion protein consisting of the extracellular domain of CTLA-4 linked to the Fc portion of human IgG1 and was approved for the treatment of RA in 2017 (Mease, 2015). It was safe and well-tolerated, and did not lead to an overall increased risk of infections, malignancies or autoimmune diseases (Ozen et al., 2019). It binds to both CD80 and CD86 on APCs with much higher affinity compared with CD28, blocking the engagement of CD28 on T cells and interfering with the T cell response and cytokine production (Zizzo et al., 2018). Abatacept can also directly modulate CD80 and CD86 expression and memory formation in human B cells (Lorenzetti et al., 2019). Phase I clinical trials showed that the application of abatacept led to clinical improvement and cytopathological reversal of psoriatic plaques in a dose-dependent manner (Abrams et al., 1999; Abrams et al., 2000). In a phase III clinical trial, abatacept treatment significantly improved patient-reported outcomes in patients with active PsA, particularly in those who were tumor necrosis factor inhibitor-naïve and/or with elevated C-reactive protein at baseline (Strand et al., 2018). However, abatacept demonstrated only a modest benefit on psoriatic skin lesions (Mease et al., 2017). Previous studies have also revealed that abatacept seems to be valuable for the treatment of PsA but less useful in the therapy of cutaneous psoriasis (Mease et al., 2011; Iannone and Lapadula, 2012). It might be due to the different dosage requirements for the optimal efficacy of abatacept for the skin vs. for arthrosis or because Th cells have common and divergent roles in the pathogenesis of psoriasis and PsA (Coates et al., 2016); thus, skin and arthrosis have different sensitivities to abatacept.

### The TIM-3:Galectin-9 Pathway

TIM-3, a vital co-inhibitory molecule of the TIM family, is specifically expressed in Th1 cells and Th17 cells but not in Th2 cells (Tang et al., 2019). To date, four ligands of TIM-3 have been discovered, including Gal-9, carcinoembryonic antigen cell adhesion molecule 1 (CEACAM-1), high-mobility group protein B1 (HMGB1), and phosphatidylserine (PtdSer) (Anderson et al., 2016). Among them, Gal-9 was the first ligand to be identified, which is a tandem-repeat type of galectin that contains two homologous carbohydrate recognition domains connected by a linker peptide. The TIM-3 and Gal-9 interaction can induce Th1 and Th17 cell apoptosis and inhibit cell differentiation and are considered to play a crucial role in immune tolerance and suppression of the T cell immune response (Hastings et al., 2009).

#### The Role of the TIM-3:Gal-9 Pathway in Psoriasis

*In vitro* studies have shown that blocking TIM-3 resulted in enhanced production of IFN-γ and IL-17 from CD4^+^ T cells. IFN-γ programs APCs to induce IL-17^+^ T cells *via* IL-1 and IL-23 secretion, supports chemokine ligand CCL20 and BD-2 production by keratinocytes synergistically with IL-17 and upregulates Gal-9 expression (Kanai et al., 2012). The level of Gal-9 in the serum of patients with psoriasis was significantly increased, while it was not associated with the pathology and severity of psoriasis (Nofal et al., 2019). It may be a part of a negative feedback mechanism. Kanai et al. found that patients with psoriasis had higher numbers of T cells producing IL-17 (Th17/Tc17 cells) or IFN-γ (Th1/Tc1 cells) than healthy donors, while they could not express TIM-3 effectively after activation (Kanai et al., 2012).

#### Targeting the TIM-3:Gal-9 Pathway for the Treatment of Psoriasis

Niwa et al. developed a stable form of galectin-9 (sGal-9) by partial deletion of the linker peptide. They reported that administration of sGal-9 markedly reduced epidermal hyperplasia and dermal cellular infiltration induced by IL-23 in the ear lobes of mice. Local levels of cytokines associated with psoriasis, such as IL-17, IL-22, IL-6, and TNF-α, were also reduced after sGal-9 treatment. In addition, the expression of activated phospho-signal transducers and activators of transcription 3 (STAT3) in epidermal keratinocytes was inhibited by sGal-9. This prompts us to conclude that sGal-9 may be a unique and useful tool for treating Th1/Th17-mediated skin inflammation, including psoriasis, which is probably mediated by the Gal-9-TIM-3 interaction (Niwa et al., 2009).

### The B-Lymphocyte and T-Lymphocyte Attenuator/CD160:Herpes Virus-Entry Mediator Pathway

With the deepening of research, we should not rule out the possibility that additional co-signaling molecules and ligands, or novel function and ligands for known molecules, remain to be discovered. For instance, the BTLA/CD160:HVEM co-inhibitory pathway has become the new research focus in recent years. HVEM (TNFRSF14, CD270) is widely expressed in both hematopoietic and non-hematopoietic cells and has a complex function. BTLA, a receptor of IgSF, is expressed on the cell membrane of most lymphoid hematopoietic cells, and CD160, a newly discovered receptor of HVEM, is mainly expressed on the surface of cytotoxic cells and T cells (Rodriguez-Barbosa et al., 2019). The combination of HVEM with BTLA or CD160 can attenuate T cell receptor-mediated signal transduction and inhibit T cell activation, but when HVEM binds to LIGHT or LTalpha, the co-stimulatory ligands of HVEM, it can stimulate the activation of T cells and stimulate the immune response of the host (Rio et al., 2009). Therefore, HVEM has dual-functional activity, but mainly negative regulation mediated by BTLA and CD160 (Rodriguez-Barbosa et al., 2019).

#### The Role of the BTLA/CD160:HVEM Pathway in Psoriasis

Recent study showed that the gene expression of CD160 and BTLA was significantly lower in psoriasis patients with health control (Youssef et al., 2019; Li et al., 2021). Another study reported that CD160 acts as a co-activator receptor for CD3-induced proliferation of CD4^+^CD160^+^ T cells isolated from psoriatic lesions (Abecassis et al., 2007). However, the mechanism that CD160/BTLA pathway acts as a role in the pathogenesis of psoriasis is not clear. Therefore, adding more insight to the mechanisms of co-signaling molecule in psoriasis may help to establish a basis for novel treatment strategies and provide new and more effective therapeutic option.

## Conclusion

In this review, we have discussed the different role of co-signaling molecules in psoriasis and the status of developing drugs targeting these co-signaling molecules. These co-signaling molecules not only have differential expressions in patients with psoriasis compared with healthy controls, but also are associated with disease severity, which might serve as potential biomarkers for psoriasis. Several biologics targeting the co-signaling molecules have shown promising outcomes for psoriasis patients, more clinical trials still need to be conducted to assess the long-term efficacy and side effects. For instance, efalizumab, a humanized mAb against LFA-1, has shown some efficacy in the preclinical study and early stage of clinical trials for psoriasis treatment, but it has been halted because of the risk of progressive multifocal leukoencephalopathy. In the future, the safeties of these new drugs targeting co-signaling molecules should be paid more attention and whether it is feasible to reduce the incidence of adverse reactions by targeting co-signaling molecules on specific cell types associated with psoriasis is worth exploring. In addition to individual applications, combining multiple biologics that target different co-stimulatory pathways and co-inhibitory pathways in psoriasis can be tested in the future.
